# Comment on: A Novel Mendelian Randomization Method With Binary Risk Factor and Outcome

**DOI:** 10.1002/gepi.70049

**Published:** 2026-06-23

**Authors:** Sandeep Chowdary Vejandla, Mengchen Ding, Hemant K. Tiwari

**Affiliations:** ^1^ Department of Biostatistics University of Alabama at Birmingham Birmingham Alabama USA

Dear Editor,

1

We write regarding the article by Allman et al., “A Novel Mendelian Randomization Method With Binary Risk Factor and Outcome,” published in *Genetic Epidemiology*, 2021:45(5):540–560. The paper addressed an important Mendelian randomization setting in which both the risk factor and outcome are binary. In continuing this methodological work, we re‐examined the mathematical formulation and computational implementation of the methods. We have identified and rectified three methodological issues: an optimization issue in the generalized method of moments (GMM) implementation, a derivation issue in the Instrumental Variable–Multivariate Bernoulli (IV‐MVB) formulation, and a simulation‐evaluation issue in the definition of bias and coverage. These corrections improve numerical stability, clarify the mathematical presentation of the methods, and provide a more appropriate evaluation of simulation performance.

Below, we outline these issues using the notation from the published manuscript, followed by our proposed corrections.

## 2 GMM Method Optimization Issue

**The Issue:**

In Allman et al., the objective function for the GMM is defined in Equation (10) as:

$$f\left(\beta_{\text{GMM}}|\theta \right)={\left(Z^{\prime }\left(Y-\text{expit}\left(X\beta_{\text{GMM}}\right)\right)\right)}^{\prime }\Omega_{\theta }^{-1}\left(Z^{\prime }\left(Y-\text{expit}\left(X\beta_{\text{GMM}}\right)\right)\right)\;\;(10)$$

The manuscript specifies the covariance matrix as $\Omega_{\theta }=Z^{\prime }\left(\theta \cdot I_{n\times n}\right)Z$, where θ is introduced as a nuisance parameter.

By substituting $\Omega_{\theta }=Z^{\prime }\left(\theta \cdot I_{n\times n}\right)Z$ into Equation (10), the objective function simplifies mathematically to:

$$f\left(\beta_{\text{GMM}}|\theta \right)=\frac{1}{\theta }{\left(Z^{\prime }\left(Y-\text{expit}\left(X\beta_{\text{GMM}}\right)\right)\right)}^{\prime }{\left(Z^{\prime }Z\right)}^{-1}\left(Z^{\prime }\left(Y-\text{expit}\left(X\beta_{\text{GMM}}\right)\right)\right).$$

Because θ factors out as a scalar denominator, it does not affect the location of the causal parameter estimate, $\hat{\beta_{\text{GMM}}}.$ However, if optimization is performed jointly over $\beta_{\text{GMM}}$ and θ, the derivative with respect to θ is strictly negative.

$$\frac{\partial f\left(\beta_{\text{GMM}}\prime \theta \right)}{\partial \theta }=-\frac{Q\left(\beta_{\text{GMM}}\right)}{\theta^{2}}<0,$$

where$\;Q\left(\beta_{\text{GMM}}\right)={\left(Z^{\prime }\left(Y-\text{expit}\left(X\beta_{\text{GMM}}\right)\right)\right)}^{\prime }{\left(Z^{\prime }Z\right)}^{-1}\left(Z^{\prime }\left(Y-\text{expit}\left(X\beta_{\text{GMM}}\right)\right)\right).$

Consequently, the optimizer is artificially driven to increase θ→∞ to reduce the objective function, rather than optimize solely over the meaningful causal parameters. This introduces an ill‐posed optimization problem with an unidentified scale direction, leading to severe convergence failures in the GMM method, as reflected in the published simulation results in Supporting Information S1: Tables 2.1 and 2.2. Furthermore, the standard errors become contaminated because the unidentified θ is subsequently plugged into the sandwich variance estimator in Equation (11).

**The Proposed Correction:**

The nuisance parameter θ must be removed entirely from the objective function and the variance estimator. The correct approach is to directly minimize:

$$Q\left(\beta_{\text{GMM}}\right)=g_{n}{\left(\beta_{\text{GMM}}\right)}^{\prime }Wg_{n}\left(\beta_{\text{GMM}}\right),$$

where

$$g_{n}\left(\beta_{\text{GMM}}\right)=\frac{1}{n}Z^{\prime }\left(Y-\text{expit}\left(X\beta_{\text{GMM}}\right)\right),W={\left(\frac{1}{n}Z^{\prime }Z\right)}^{-1}.$$

This preserves the GMM estimating principle while optimizing only over the causal parameter vector. By optimizing exclusively over the meaningful causal parameters ($\beta_{\text{GMM}}$), the problem becomes properly identified and numerically stable. After this correction, GMM convergence improved substantially, particularly in weak‐ and moderate‐instrument settings (e.g., from roughly 100 convergences in the published results to over 4000 successful convergences out of 8000 simulations), while the bias estimates remained broadly consistent when convergence was achieved.

## 3 IV‐MVB Method Derivation Corrections

**The Issue:**

In Section 2.2, Allman et al. use the expression

$$\rho =\text{Corr}(X,Y)=\theta \sqrt{\text{Var}(X)\text{Var}(Y)}.$$

This expression mischaracterizes θ as the covariance, Cov(X,Y).

If θ=Cov(X,Y), then the standard relationship is

$$\rho =\frac{\text{Cov}(X,Y)}{\sqrt{\text{Var}(X)\text{Var}(Y)}}=\frac{\theta }{\sqrt{\text{Var}(X)\text{Var}(Y)}}.$$

**The Proposed Correction:**

For binary $X_{i}$ and $Y_{i}$, the standard relationship between correlation and covariance is:

$$\rho =\frac{\text{Cov}\left(X_{i},Y_{i}\right)}{\sqrt{\text{Var}\left(X_{i}\right)\text{Var}\left(Y_{i}\right)}}=\frac{p_{11i}-p_{X_{i}}p_{Y_{i}}}{\sqrt{p_{X_{i}}p_{Y_{i}}\left(1-p_{X_{i}}\right)\left(1-p_{Y_{i}}\right)}}$$

Solving for $p_{11i}$ yields:

$$p_{11i}=P\left(X_{i}=1,Y_{i}=1\right)=p_{X_{i}}p_{Y_{i}}+\rho \sqrt{p_{X_{i}}p_{Y_{i}}\left(1-p_{X_{i}}\right)\left(1-p_{Y_{i}}\right)}$$

The remaining joint probabilities ($p_{10i}$, $p_{01i}$, $p_{00i}$) follow directly from $p_{11i}$. To simplify these expressions for computational implementation, θ must be formally re‐defined and interpreted as a “scaled association parameter” rather than covariance:

$$\theta =\frac{\rho }{\sqrt{\text{Var}\left(X_{i}\right)\text{Var}\left(Y_{i}\right)}}=\frac{\rho }{\sqrt{p_{X_{i}}\left(1-p_{X_{i}}\right)p_{Y_{i}}\left(1-p_{Y_{i}}\right)}}.$$

Then the joint probabilities are correctly written as

$$p_{11i}=p_{X_{i}}p_{Y_{i}}\{1+\theta (1-p_{X_{i}})(1-p_{Y_{i}})\},$$

$$p_{10i}=p_{X_{i}}(1-p_{Y_{i}})\{1-\theta (1-p_{X_{i}})p_{Y_{i}}\},$$

$$p_{01i}=(1-p_{X_{i}})p_{Y_{i}}\{1-\theta p_{X_{i}}(1-p_{Y_{i}})\},$$

and

$$p_{00i}=(1-p_{X_{i}})(1-p_{Y_{i}})\{1+\theta p_{X_{i}}p_{Y_{i}}\}.$$

This correction appears to be primarily mathematical rather than computational. Re‐estimation under the corrected IV‐MVB formulation did not produce meaningful deviations from the published numerical results, suggesting that the underlying implementation may have already used the correct expressions and parameterization. Therefore, for the IV‐MVB method, the issue appears to lie in how the derivation was presented in the published manuscript rather than in the original computational implementation.

## 4 Bias and Coverage Evaluation in the Simulation Study

**The Issue:**

In Section 2.3, Allman et al. state that each MR method was evaluated “in terms of their bias in estimating the causal parameter $\beta_{1}$.” The published bias expression was defined as

$$\text{bias}({\hat{\beta }}_{i})=\beta_{i,\text{true}}-{\hat{\beta }}_{i},$$

where $\beta_{i,\text{true}}$ was obtained from the “true” model, controlling for the simulated confounder $U_{i}$. In other words, for each simulated dataset, the MR estimate $\hat{\beta_{i}}$ was compared against a separate fitted estimate from the correctly specified model that included the simulated confounder $U_{i}$.

The issue is that, in the simulation itself, the outcome was generated from the fixed data‐generating model

$$\text{logit}\{P(Y_{i}=1|X_{i},U_{i})\}=\beta_{0}+\beta_{1}X_{i}+c_{Y}U_{i}.$$

Therefore, the actual causal parameter being targeted is already the fixed simulation parameter $\beta_{1}$. Fitting a separate “true” model within each simulated dataset produces a replicate‐specific estimate, $\beta_{i,\text{true}}$, rather than the fixed simulation truth. As a result, the reported bias partly reflects random variation from this additional fitted model, instead of measuring only the bias of the MR estimator relative to the known data‐generating causal parameter.

**The Proposed Correction:**

Bias should be evaluated relative to the fixed causal parameter used to generate the data. Using the estimate‐minus‐truth convention, the corrected bias expression is

$$\text{bias}({\hat{\beta }}_{i})={\hat{\beta }}_{i}-\beta_{1}.$$

Similarly, coverage should be assessed by whether the confidence interval for $\hat{\beta_{i}}$ contains the fixed causal parameter $\beta_{1}$, rather than a replicate‐specific estimate from the fitted “true” model. This correction did not lead to meaningful differences between the original and corrected simulation summaries, but it is warranted because it aligns the evaluation of bias and coverage with the known data‐generating parameter in the simulation study.

We also re‐implemented the simulation study following the original design and compared Wald, 2SPS, 2SRI, GMM, and IV‐MVB after incorporating these corrections. The corrected implementation improves numerical stability and reproducibility, especially for GMM, while the corrected bias and coverage definitions align the simulation summaries with the fixed data‐generating causal parameter. Overall, these changes preserve the central conclusion that IV‐MVB performs comparably to two‐stage methods and provides a coherent likelihood‐based framework for inference with binary risk factors and binary outcomes. Updated R code, simulation summaries, tables, supplementary tables, and figures are available at GitHub: https://github.com/sding10/Mendelian-randomization.

We hope these clarifications serve as a useful addendum to this novel methodology, ensuring its mathematical robustness and practical implementation for future researchers.

## Conflicts of Interest

The authors declare no conflicts of interest.

## Supporting information

Supporting File

## Data Availability

The data that support the findings of this study are openly available in GitHub at https://github.com/sding10/Mendelian-randomization.

