# Supplementary material for: Comment on: A Novel Mendelian Randomization Method With Binary Risk Factor and Outcome
Source: Genet Epidemiol. 2026 Jun 23;50(5):e70049. doi: 10.1002/gepi.70049 (PMC13288442; doi:10.1002/gepi.70049)
Supplement: Supplementary file 1 — Supporting File [file GEPI-50-0-s001.docx]

**Supplementary Table 2.1**. Results from Monte Carlo simulations comparing bias in causal parameter estimates from the four existing MR methods and the proposed method: Scenario’s A and B as a function of instrument strength.

| **Instrument Strength (**$\boldsymbol{\alpha}_{\boldsymbol{1}}$**)** | **MR Method** | **Scenario A** | | **Scenario B** | |
| --- | --- | --- | --- | --- | --- |
|  |  | **Num Converged** | **Median (Q_1_, Q_3_) Bias** | **Num Converged** | **Median (Q_1_, Q_3_) Bias** |
| 0.01 | Wald | 7991 | 0.12 (-0.86, 1.12) | 7990 | -0.65 (-1.62, 0.35) |
|  | 2SPS | 7991 | 0.48 (-3.45, 4.48) | 7990 | 0.38 (-3.49, 4.41) |
|  | 2SRI | 8000 | 0.48 (-3.49, 4.52) | 8000 | 0.45 (-3.86, 4.91) |
|  | GMM | 3809 | 0.17 (-1.47, 1.85) | 6482 | -0.09 (-3.76, 6.99) |
|  | IV-MVB | 7946 | -0.03 (-4.51, 3.53) | 7919 | -0.82 (-6.71, 2.49) |
| 0.1 | Wald | 7986 | 0.08 (-0.76, 0.92) | 7988 | -0.71 (-1.57, 0.12) |
|  | 2SPS | 7986 | 0.33 (-3.07, 3.71) | 7988 | 0.14 (-3.30, 3.48) |
|  | 2SRI | 8000 | 0.34 (-3.10, 3.75) | 8000 | 0.18 (-3.67, 3.88) |
|  | GMM | 4195 | 0.17 (-1.42, 1.76) | 6564 | -0.28 (-3.58, 3.51) |
|  | IV-MVB | 7929 | -0.03 (-3.80, 3.03) | 7907 | -0.78 (-5.34, 2.06) |
| 0.2 | Wald | 7996 | 0.04 (-0.58, 0.62) | 7997 | -0.74 (-1.33, -0.15) |
|  | 2SPS | 7996 | 0.16 (-2.31, 2.49) | 7997 | 0.04 (-2.33, 2.44) |
|  | 2SRI | 8000 | 0.15 (-2.36, 2.51) | 8000 | 0.06 (-2.58, 2.72) |
|  | GMM | 5091 | 0.14 (-1.26, 1.54) | 6793 | -0.28 (-2.49, 2.00) |
|  | IV-MVB | 7959 | -0.00 (-2.61, 2.19) | 7927 | -0.43 (-3.30, 1.72) |
| 0.3 | Wald | 7998 | 0.01 (-0.44, 0.42) | 7999 | -0.77 (-1.19, -0.38) |
|  | 2SPS | 7998 | 0.04 (-1.78, 1.68) | 7999 | -0.08 (-1.75, 1.50) |
|  | 2SRI | 8000 | 0.03 (-1.81, 1.71) | 8000 | -0.08 (-1.95, 1.68) |
|  | GMM | 6180 | 0.07 (-1.27, 1.32) | 7151 | -0.29 (-1.88, 1.25) |
|  | IV-MVB | 7984 | -0.03 (-1.90, 1.57) | 7974 | -0.23 (-2.00, 1.32) |
| 0.5 | Wald | 8000 | 0.00 (-0.25, 0.25) | 8000 | -0.78 (-1.02, -0.54) |
|  | 2SPS | 8000 | -0.00 (-1.01, 1.01) | 8000 | -0.08 (-1.09, 0.88) |
|  | 2SRI | 8000 | -0.00 (-1.03, 1.01) | 8000 | -0.10 (-1.22, 0.98) |
|  | GMM | 7523 | 0.02 (-0.93, 0.98) | 7635 | -0.15 (-1.14, 0.84) |
|  | IV-MVB | 7998 | -0.00 (-1.02, 1.00) | 7998 | -0.09 (-1.10, 0.86) |
| 0.7 | Wald | 8000 | 0.00 (-0.18, 0.17) | 8000 | -0.78 (-0.95, -0.61) |
|  | 2SPS | 8000 | 0.01 (-0.73, 0.72) | 8000 | -0.09 (-0.80, 0.62) |
|  | 2SRI | 8000 | 0.01 (-0.75, 0.72) | 8000 | -0.11 (-0.90, 0.67) |
|  | GMM | 7922 | 0.02 (-0.73, 0.72) | 7907 | -0.10 (-0.80, 0.64) |
|  | IV-MVB | 8000 | 0.01 (-0.73, 0.72) | 8000 | -0.09 (-0.80, 0.62) |
| 1.0 | Wald | 8000 | -0.00 (-0.12, 0.12) | 8000 | -0.78 (-0.90, -0.66) |
|  | 2SPS | 8000 | -0.00 (-0.53, 0.51) | 8000 | -0.08 (-0.59, 0.43) |
|  | 2SRI | 8000 | -0.01 (-0.54, 0.51) | 8000 | -0.11 (-0.68, 0.45) |
|  | GMM | 7997 | -0.00 (-0.53, 0.51) | 7996 | -0.08 (-0.59, 0.45) |
|  | IV-MVB | 8000 | 0.00 (-0.53, 0.51) | 8000 | -0.08 (-0.60, 0.43) |

**Supplementary Table 2.2**. Results from Monte Carlo simulations comparing bias in causal parameter estimates from the four existing MR methods and the proposed method: Scenario’s C and D across various sample sizes.

| **Sample Size** | **MR**  **Method** | **Scenario C** | | **Scenario D** | |
| --- | --- | --- | --- | --- | --- |
|  |  | **Num Converged** | **Median (Q_1_, Q_3_) Bias** | **Num Converged** | **Median (Q_1_, Q_3_) Bias** |
| 50 | Wald | 7878 | 0.03 (-0.64, 0.77) | 7890 | -0.76 (-1.36, 0.00) |
|  | 2SPS | 7878 | 0.23 (-2.87, 3.34) | 7890 | 0.15 (-2.65, 3.39) |
|  | 2SRI | 8000 | 0.25 (-2.89, 3.36) | 8000 | 0.13 (-2.99, 3.70) |
|  | GMM | 6372 | 0.10 (-2.37, 2.73) | 6697 | -0.21 (-2.67, 2.89) |
|  | IV-MVB | 7384 | 0.05 (-3.17, 3.13) | 6793 | -0.23 (-3.31, 2.46) |
| 200 | Wald | 7992 | 0.00 (-0.40, 0.38) | 7994 | -0.77 (-1.17, -0.38) |
|  | 2SPS | 7992 | 0.02 (-1.65, 1.63) | 7994 | -0.00 (-1.72, 1.62) |
|  | 2SRI | 8000 | 0.01 (-1.69, 1.64) | 8000 | -0.03 (-1.94, 1.76) |
|  | GMM | 6705 | 0.03 (-1.37, 1.35) | 7018 | -0.27 (-1.88, 1.17) |
|  | IV-MVB | 7982 | -0.00 (-1.73, 1.57) | 7960 | -0.13 (-1.88, 1.45) |
| 500 | Wald | 8000 | 0.00 (-0.25, 0.25) | 8000 | -0.78 (-1.03, -0.54) |
|  | 2SPS | 8000 | 0.02 (-1.02, 1.04) | 8000 | -0.09 (-1.14, 0.94) |
|  | 2SRI | 8000 | 0.01 (-1.04, 1.05) | 8000 | -0.12 (-1.28, 1.02) |
|  | GMM | 7510 | 0.03 (-0.93, 1.01) | 7606 | -0.17 (-1.20, 0.86) |
|  | IV-MVB | 7999 | 0.01 (-1.03, 1.03) | 7998 | -0.10 (-1.15, 0.92) |
| 1000 | Wald | 8000 | -0.00 (-0.18, 0.17) | 8000 | -0.78 (-0.96, -0.61) |
|  | 2SPS | 8000 | -0.01 (-0.75, 0.69) | 8000 | -0.10 (-0.82, 0.62) |
|  | 2SRI | 8000 | -0.02 (-0.76, 0.69) | 8000 | -0.12 (-0.92, 0.67) |
|  | GMM | 7924 | -0.01 (-0.74, 0.69) | 7896 | -0.11 (-0.83, 0.63) |
|  | IV-MVB | 8000 | -0.02 (-0.75, 0.68) | 8000 | -0.10 (-0.82, 0.61) |
| 1500 | Wald | 8000 | -0.00 (-0.14, 0.14) | 8000 | -0.78 (-0.92, -0.64) |
|  | 2SPS | 8000 | -0.01 (-0.59, 0.58) | 8000 | -0.08 (-0.68, 0.48) |
|  | 2SRI | 8000 | -0.01 (-0.61, 0.59) | 8000 | -0.11 (-0.77, 0.51) |
|  | GMM | 7985 | -0.01 (-0.60, 0.59) | 7978 | -0.08 (-0.69, 0.51) |
|  | IV-MVB | 8000 | -0.01 (-0.59, 0.58) | 8000 | -0.09 (-0.69, 0.48) |
| 2000 | Wald | 8000 | -0.00 (-0.13, 0.12) | 8000 | -0.78 (-0.90, -0.66) |
|  | 2SPS | 8000 | -0.01 (-0.52, 0.50) | 8000 | -0.07 (-0.58, 0.42) |
|  | 2SRI | 8000 | -0.01 (-0.53, 0.50) | 8000 | -0.09 (-0.65, 0.45) |
|  | GMM | 7999 | -0.01 (-0.52, 0.50) | 7998 | -0.07 (-0.58, 0.45) |
|  | IV-MVB | 8000 | -0.00 (-0.52, 0.50) | 8000 | -0.08 (-0.58, 0.41) |
| 2500 | Wald | 8000 | 0.00 (-0.11, 0.11) | 8000 | -0.78 (-0.89, -0.67) |
|  | 2SPS | 8000 | 0.01 (-0.46, 0.46) | 8000 | -0.08 (-0.54, 0.35) |
|  | 2SRI | 8000 | 0.01 (-0.46, 0.46) | 8000 | -0.11 (-0.61, 0.38) |
|  | GMM | 7997 | 0.01 (-0.46, 0.47) | 7997 | -0.08 (-0.54, 0.38) |
|  | IV-MVB | 8000 | 0.01 (-0.45, 0.46) | 8000 | -0.09 (-0.54, 0.35) |
